# Effect of yellowing time on bioactive compounds in yellow tea and their antiproliferative capacity in HepG2 cells

**DOI:** 10.1002/fsn3.1036

**Published:** 2019-04-18

**Authors:** Ran An, Lingli Sun, Limin Xiang, Wenji Zhang, Qiuhua Li, Xingfei Lai, Shuai Wen, Mengen Huo, Dongli Li, Shili Sun

**Affiliations:** ^1^ School of Biotechnology and Health Sciences Wuyi University Jiangmen China; ^2^ Guangdong Academy of Agricultural Sciences/Guangdong Key Laboratory of Tea Resources Innovation & Utilization Tea Research Institute Guangzhou China; ^3^ International Healthcare Innovation Institute (Jiangmen) Jiangmen China

**Keywords:** anticancer, antioxidation, bioactive component, yellow tea, yellowing time

## Abstract

Several studies have shown potent antineoplastic effects of tea, which can induce apoptosis and inhibit proliferation of cancer cells. Yellow tea is one of the six major types of tea, and yellowing time, a key factor in its processing, is known to improve its quality and bioactivity. However, the effects of yellowing on the composition of the bioactive substances of tea are poorly understood. We analyzed the biochemical composition and the antioxidant and anticancer activities of the extracts of yellow tea (EYTs) subjected to different yellowing durations. Prolonged yellowing increased the content of water extracts, amino acids, soluble sugars, theaflavins, and nonesterified catechins (*p < *0.05, *p* < 0.01) and decreased that of polyphenols, flavonols, thearubigins, caffeine, GA, and esterified catechins (*p < *0.05, *p* < 0.01). In addition, yellowing also slightly increased the antioxidant capacity of the EYTs, but did not significantly affect their ability to inhibit the proliferation of the hepatocarcinoma HepG2 cells. Mechanistically, the EYTs significantly downregulated the phosphorylation of PI3K and AKT and upregulated the Bax/Bcl‐2 ratio in the HepG2 cells. Taken together, the yellowing time influences the bioactive components of yellow tea, and the resulting yellow tea may have more potent antioxidant and anticancer effects.

## 1 INTRODUCTION

Tea is the second most consumed nonalcoholic beverage in the world, next only to water (Xu et al., 2018). There are six main kinds of tea—green, white, yellow, black, oolong, and dark—which differ in their processing, flavor, and aroma. In addition, there are some rare kinds of processed tea, such as the Pu'er tea which is produced in southwestern Yunnan. Despite differences in processing, the method of drinking tea is similar, that is, steeping it in hot water for few minutes. In addition, tea is associated with potent anticancer, antioxidative, and anti‐inflammatory effects, which may differ depending on the type of tea (Singh, Rawat, Bhagat, & Singh, 2017; Teng, Li, Guruvaiah, Xu, & Xie, 2018). Yellow tea is similar to green tea, but has higher total soluble sugar content due to an additional yellowing or fermentation step, which removes the characteristic grassy smell and bestows a sweet, fruity, and floral aroma, while preserving the health benefits of green tea (Bian et al., 2016; Kujawska et al., 2016; Teng et al., 2018; Xu et al., 2018).

Several kinds of tea have strong antioxidant activity, and their extracts can scavenge free radicals like 2,2‐diphenyl‐1‐picrylhydrazyl (DPPH) and hydroxyl ions (OH) (Chen, Liao, Hsu, Tsai, & Hsueh, 2018; Kmiecik, Gramza‐Micha Owska, & Korczak, 2018). In addition, the antineoplastic effects of black tea, oolong tea, white tea, green tea, and Pu'er tea are well documented, while little is known regarding the anticancer effects of yellow tea (Zhou et al., 2017).

The aims of our study were to identify the bioactive components in Yinghong NO.9 yellow tea (YT) relative to the yellowing time and to assess their biological effects on a liver cancer cell line. Yinghong NO.9 yellow tea is very similar to green tea, except for the additional yellowing process. The fresh leaves (a bud with two leaves) are first harvested, and withered for at least 4–6 hr to reduce their water content to 60%–70%. The leaves are then heated at 150–200°C to remove the grassy smell. After cooling to ambient temperature, the leaves are slightly twisted to bruise the, and heaped to yellow for 0–16 hr, followed by baking and dehydration.

## 2 MATERIALS AND METHODS

### 2.1 Tea samples and reagents

Yinghong NO.9 samples yellowed for 0 hr (YT0h), 4 hr (YT4h), 8 hr (YT8h), 12 hr (YT12h), and 16 hr (YT16h) were provided by the Tea Research Institute, Guangdong Academy of Agricultural Sciences. ABTS and DPPH were obtained from Yuanye Biotechnology Co. Ltd. and TPTZ from MYM Biotechnology Co. Ltd. All catechins used for HPLC were obtained from Reference Research and Folin–Ciocalteu's phenol reagent from Solarbio Science & Technology Co. Ltd. All other chemical reagents were analytically pure and purchased from Damao Chemical Reagent Factory. Water was double‐distilled from the Laboratory Water Purification System (Ewell Bio‐Technology Co. Ltd.).

### 2.2 Preparation and analysis of extracts of YT (EYTs)

The powder prepared from YT samples was extracted by boiling in double‐distilled water thrice for 30 min each (tea/water, 1:20, w:v). The aqueous extracts were pooled, centrifuged, concentrated at 60°C, and finally lyophilized in lyophilizer (Storge Box, Beijing, China).

The bioactive component contents of the EYTs were determined according to the GB/T8304‐2002 guidelines. The water content was measured in terms of the weight difference before and after heating at 130°C for 3 hr. The content of free amino acids was determined by the ninhydrin methods: Briefly, 1 g of each EYT was dissolved in 90 ml boiled water, and after mixing 1 ml of the solution with 0.5 ml PBS (pH = 8) and 0.5 ml ninhydrin at 100°C for 15 min, the absorbance was measured at 570 nm (Troll & Cannan, 1953). The anthrone–sulfuric acid colorimetric assay was used to determine the total soluble sugar content: Briefly, 100 mg anthrone was dissolved in 100 ml H_2_SO_4_, and 8 ml of this solution was mixed with 1 ml EYT solution (as prepared above) at 100°C for 3 min, followed by detection of absorbance at 620 nm. Tea polyphenols were measured as described by Masuko et al. (Ng et al., 2009). Briefly, 0.2 g EYTs was extracted twice with 5 ml 70% (v/v) methanol, mixed with 5 ml 10% Folin's phenol (v/v) for 3–8 min, and reacted with 4 ml 7% NaCO_3_ at room temperature for 60 min before measuring the absorbance at 765 nm.

To analyze the catechins, the epicatechin (EC), catechin (C), epicatechin gallate (ECG), gallocatechin (GC), epigallocatechin gallate (EGCG), gallocatechin gallate (GCG), catechin gallate (CG), epigallocatechin (EGC), gallic acid (GA), and caffeine (CAFF) standards were first run on an Agilent HPLC column (5 μm, 250 × 4.6 mm) using an HPLC system (Agilent Technologies Ltd.), according to a modified version of the method of Zuo et al. (Cabrera, Giménez, & López, 2003). The tea samples were prepared by extracting 0.2 g twice with 5 ml 70% (v/v) methanol at 70°C for 10 min, and filtering through a 0.45‐μm nylon membrane (Millipore), and 10 µl of each sample was injected into the HPLC system. A gradient elution was performed at the flow rate of 1 ml/min, with each run starting with 100% solvent A (double‐distilled water) for 10 min, followed by solvent B (methanol), solvent C (0.05% phosphate acid v/v), and solvent D (acetonitrile). The wavelength was set in the range of 200–400 nm. The described technique is routinely used in the quality control process during tea manufacturing (Cabrera et al., 2003).

### 2.3 Antioxidant activity assay

The free radical scavenging potential of the EYTs was evaluated using DPPH as previously described (Chen et al., 2018; Kmiecik et al., 2018). Briefly, 1 ml of each EYT solution or ethanol (blank) was mixed with 2 ml of 0.1 mM DPPH (in ethanol), and the absorbance was measured at 515 nm. The total antioxidation capacity was measured by the ferric reducing ability of plasma (FRAP) assay. The FRAP reagent was prepared by mixing 25 ml of 300 mM acetate buffer (pH 3.6), 2.5 ml of 10 mM TPTZ (2,4,6‐tripyridyl‐s‐triazine in HCl), and 2.5 ml of 20 mM FeCl_3_·6H_2_O. The FRAP reagent was then mixed with different concentrations of each EYT sample or blank (water) at the ratio of 30:1, and the absorbance was measured at 593 nm (Benzie & Strain, 1996). To detect OH radical clearance, 1 ml of each sample or blank (water) was mixed with 2 ml of 1.8 mM FeSO_4_, 1.5 ml of 1.8 mM salicylic acid (in ethanol), and 0.1 ml of 0.3% H_2_O_2_, and the absorbance was detected at 510 nm (Brand‐Williams, Cuvelier, & Berset, 1995). Furthermore, the clearance of 2,2′‐azino‐bis(3‐ethylbenzothiazoline‐6‐sulfonic acid) or ABTS was determined as described by Friedman et al. (Tang & Liu, 2007). Briefly, 7 mM ABTS and 2.45 mM potassium peroxydisulfate was mixed with EYTs or water (blank) and the absorbance was detected at 734 nm. The clearance of all free radicals was expressed as D (%) = [(A_0_ − A_S_)/A_0_] * 100% (A_0_—control; and A_S_—sample).

### 2.4 Cell culture and viability assay

Human hepatoblastoma HepG2 cells were obtained from the Cell Resources Center of Shanghai Academy of Sciences, Chinese Academy of Sciences, and cultured in DMEM (Gibco by Life Technology) with 10% (v/v) fetal bovine serum (FBS) (Tianhang Biotechnology Ltd.) and 1% penicillin/streptomycin (Gibco by Life Technology) at 37°C in a humidified incubator (5% CO_2_ and 95% air).

For MTT assay, the HepG2 cells were seeded into 96‐well plates at the density of 5,000 cells/well and, after 24 hr, treated with varying concentrations (0, 0.125, 0.25, 0.5, 1 mg/ml) of the different EYTs (dissolved in serum‐free DMEM) for another 24 hr. After replenishing each well with 90 μl fresh medium, 10 μl MTT solution (MYM Biotechnology, Co., Ltd.) was added at the final concentration of 0.5 mg/ml per well. The cells were further incubated for 4 hr, and the reaction was stopped by adding 150 μl DMSO (Biosharp) per well. The absorbance at 490 nm was measured by plate reader fitted with a spectrophotometer (BERTHOLD Technologies). Taking viability of the control (DMSO‐treated) cells as 100%, that of the YET‐treated cells was calculated as V (%) = (A_s_ − A_b_)/(A_c_ − A_b_) * 100% (A_s_—absorbance of samples; A_b_—absorbance of DMSO‐treated cells; and A_c_—absorbance of untreated control cells).

### 2.5 Western blotting

Total protein was extracted from differentially treated HepG2 cells using RIPA buffer supplemented with 1% PMSF (Beyotime) at 4°C. Equal amounts of protein per sample were separated by 10% SDS‐PAGE and then transferred onto polyvinylidene difluoride membranes. The membranes were blocked with 10% nonfat milk for 90 min and then incubated overnight with primary antibodies against PI3K, p‐PI3K, AKT, p‐AKT (Cell Signaling Technology), Bax, Bcl‐2 (Abcam), and β‐actin (Sigma‐Aldrich) at 4°C. After washing with TBST, the membranes were incubated with the secondary antibodies for 50 min. Chemiluminescent signals were developed using an ECL kit (Bio‐Rad) and visualized with the ChemiDoc XRS gel documentation system (Tanon). The protein bands were analyzed by ImageJ, with β‐actin as the internal control (Sun, Wang, Liu, & Wang, 2015; Sun, Zhang, et al., 2015).

### 2.6 Statistical analyses

Statistical analysis was performed using SPSS Statistics software version 13.0 (SPSS Inc.) and Prism 6.0 software for Windows (GraphPad Software). All data are presented as means ± *SD* of at least three independent experiments. Multiple groups were compared by analysis of variance (ANOVA) and Tukey's post hoc test, and Pearson's correlation coefficient (*r*) was used to determine correlation between any two parameters. *p* Values <0.05 were considered statistically significant.

## 3 RESULTS

### 3.1 Bioactive components of EYTs

Several studies have reported significant alterations in the composition of yellow tea during yellowing (Friedman, Levin, Lee, & Kozukue, 2009; Narumi et al., 2014). Therefore, we compared the EYT samples subjected to different yellowing times. As shown in Table 1, soluble sugars, free amino acids, and TFs were significantly higher in the YT12h and YT16h samples compared to YT0h, and increased content of total soluble sugar was associated with improved taste. In contrast, the levels of tea polyphenols, flavonols, and TRs decreased significantly in the YT12h and YT16h samples. Furthermore, the content of GA, total catechins, and esterified catechins gradually decreased with the yellowing time, with significantly lower levels in the YT12h and YT16h samples, while the nonesterified catechins increased markedly (Table 2). Taken together, the duration of the yellowing process significantly affects the composition of yellow tea and may potentially alter its benefits.

**Table 1:** The contents of bioactive components in Yinghong NO.9 yellow tea

Component	YT0h	YT4h	YT8h	YT12h	YT16h
Water extracts (%)	41.06 ± 0.00	41.53 ± 0.03*	42.22 ± 0.03**	44.34 ± 0.04**	44.47 ± 0.02**
Tea polyphenols (TP, %)	30.30 ± 0.16	29.33 ± 0.04	29.16 ± 0.08*	28.96 ± 0.01**	28.41 ± 0.09**
Amino acid (AA, %)	1.72 ± 0.01	1.83 ± 0.01	2.36 ± 0.00**	2.52 ± 0.02**	2.69 ± 0.01**
TP/AA	17.71 ± 0.16	16.04 ± 0.07*	12.32 ± 0.03**	11.43 ± 0.09**	10.68 ± 0.15**
Soluble sugar (%)	2.94 ± 0.03	2.99 ± 0.00	2.99 ± 0.02	3.04 ± 0.03	3.14 ± 0.02
Flavonols (mg/g)	9.30 ± 0.02	9.11 ± 0.00	9.09 ± 0.00	9.06 ± 0.03	8.86 ± 0.00
Theaflavins (TFs, %)	0.10 ± 0.01	0.11 ± 0.00	0.12 ± 0.03	0.13 ± 0.02	0.16 ± 0.00
Thearubigins (TRs, %)	3.32 ± 0.08	3.31 ± 0.06	2.80 ± 0.47**	2.74 ± 0.11**	2.74 ± 0.05*
Theabrownins (TBs, %)	1.52 ± 0.07	1.25 ± 0.11	2.00 ± 0.07*	1.82 ± 0.14*	1.75 ± 0.05

Note

Values represent means ± *SD* (*n* = 3).

* *p* < 0.05 versus the YT0h group.

** *p* < 0.01 versus the YT0h group.

**Table 2:** The contents of catechin monomers, caffeine, and gallic acid in Yinghong NO.9 yellow tea (mg/g)

Component	YT0h	YT4h	YT8h	YT12h	YT16h
GA	6.82 ± 0.01	6.73 ± 0.03	6.72 ± 0.0.01	6.69 ± 0.00	6.40 ± 0.08**
GC	7.00 ± 0.10	7.15 ± 0.02	7.16 ± 0.02	7.26 ± 0.07*	7.32 ± 0.04*
EGC	18.63 ± 0.24	20.64 ± 0.28**	21.04 ± 0.01**	21.05 ± 0.14**	21.08 ± 0.13**
C	18.32 ± 0.32	18.60 ± 0.16*	18.76 ± 0.05**	18.78 ± 0.01**	18.87 ± 0.45**
CAFF	47.03 ± 0.48	45.50 ± 0.16*	45.42 ± 0.01*	45.41 ± 0.01*	44.56 ± 0.33**
EC	27.16 ± 0.25	27.18 ± 0.03	27.99 ± 0.12**	28.04 ± 0.08**	28.90 ± 0.11**
EGCG	55.82 ± 0.78	52.76 ± 0.39**	52.72 ± 0.15**	52.49 ± 0.63**	50.23 ± 0.33**
ECG	91.03 ± 0.56	89.24 ± 0.51**	87.81 ± 0.09**	86.77 ± 0.62**	86.76 ± 0.06**
CG	1.00 ± 0.03	0.98 ± 0.01	0.74 ± 0.02*	0.68 ± 0.02*	0.63 ± 0.00*
Catechins	219.45 ± 0.64	216.84 ± 0.96*	216.12 ± 0.12*	214.68 ± 0.76*	214.20 ± 0.23**
Esterified catechins	147.39 ± 0.13	142.66 ± 0.07**	141.30 ± 0.04**	140.37 ± 0.60**	137.77 ± 0.14**
Nonesterified catechins	71.32 ± 0.27	73.41 ± 0.20**	74.88 ± 0.08**	75.05 ± 0.04**	75.92 ± 0.36**

Note

Values represent means ± *SD* (*n* = 3).

* *p* < 0.05 versus the YT0h group.

** *p* < 0.01 versus the YT0h group.

### 3.2 Correlation between yellowing time and bioactive components

As shown in Table 3, the correlation coefficients (*r*) of water extract, amino acid, and theaflavin contents with yellowing duration were 0.953 (*p* < 0.05), 0.976 (*p* < 0.01), and 0.963 (*p* < 0.01), respectively, indicating a positive correlation between these bioactive components and time. In contrast, tea polyphenols (*r *= −0.965, *p* < 0.01) and the ratio of tea polyphenols to amino acid (TP/AA; *r *= −0.972, *p* < 0.01) were negatively correlated with yellowing time. Similarly, GC (*r = *0.971, *p* < 0.01), C (*r = *0.935, *p* < 0.05), EC (*r = *0.951, *p* < 0.05), and nonesterified catechins (*r = *0.95, *p* < 0.05) were positively correlated, while CAFF (*r *= −0.889, *p* < 0.05), EGCG (*r* = −0.91, *p* < 0.05), ECG (*r* = −0.955, *p* < 0.05), CG (*r* = −0.953, *p* < 0.05), catechins (*r* = −0.963, *p* < 0.01), and esterified catechins (*r* = −0.958, *p* < 0.05) were negatively correlated with yellowing duration. Finally, no significant correlation was seen between yellowing time and EGC, GA, and soluble sugar (*p* > 0.05). These findings strongly suggested that the bioactive components of yellow tea depend on the yellowing time.

**Table 3:** Correlation coefficient (*r*) between yellowing time and bioactive components of Yinghong NO.9 yellow tea

Component	Correlation coefficient (*r*)	Component	Correlation coefficient (*r*)
Water extracts	0.953*	EGC	0.796
Tea polyphenols	−0.965**	C	0.935*
Amino acid	0.976**	CAFF	−0.889*
TP/AA	−0.972**	EC	0.951*
Soluble sugar	0.426	EGCG	−0.91*
Flavonols	−0.858	ECG	−0.955*
Theaflavins	0.963**	CG	−0.953*
Thearubigins	−0.855	Catechins	−0.963**
Theabrownins	0.454	Esterified catechins	−0.958*
GA	−0.872	Nonesterified catechins	0.95*
GC	0.971**		

Note

Correlation is significant at the following levels.

* *p* < 0.05.

** *p* < 0.01.

### 3.3 Yellowing time affects the antioxidant activity of EYTs

**Figure 1:**
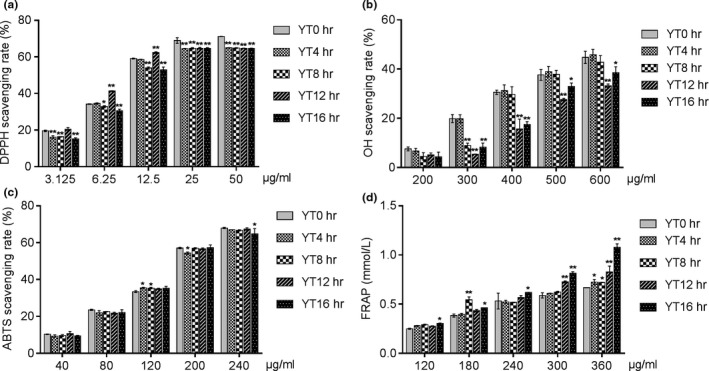
Scavenging rate of DPPH (a), scavenging rate of hydroxyl radicals (b), scavenging rate of ABTS (c), and antioxidant capacity in vitro (d). Data are presented as mean ± *SD* of three independent experiments. *p < 0.05 versus the YT0h group; **p < 0.01 versus the YT0h group

The EYTs showed significant antioxidant effects in vitro, evaluated in terms of scavenging free radicals (DPPH and OH) and the total antioxidant capacity (FRAP), in a dose‐dependent manner. Compared to YT0h (green tea), however, the free radical scavenging ability of the different EYTs decreased significantly, with little effect of the yellowing time (Figure 1a,c). Scavenging of the OH radicals decreased during the yellowing process, and the lowest clearance was seen for YT12h (Figure 1b). On the contrary, the total antioxidant capacity of the YT16h group was higher compared to the other samples (Figure 1d). Taken together, EYTs show appreciable antioxidant activity, which is affected to some extent by the yellowing duration.

### 3.4 EYTs inhibit HepG2 cell proliferation independent of yellowing time

**Figure 2:**
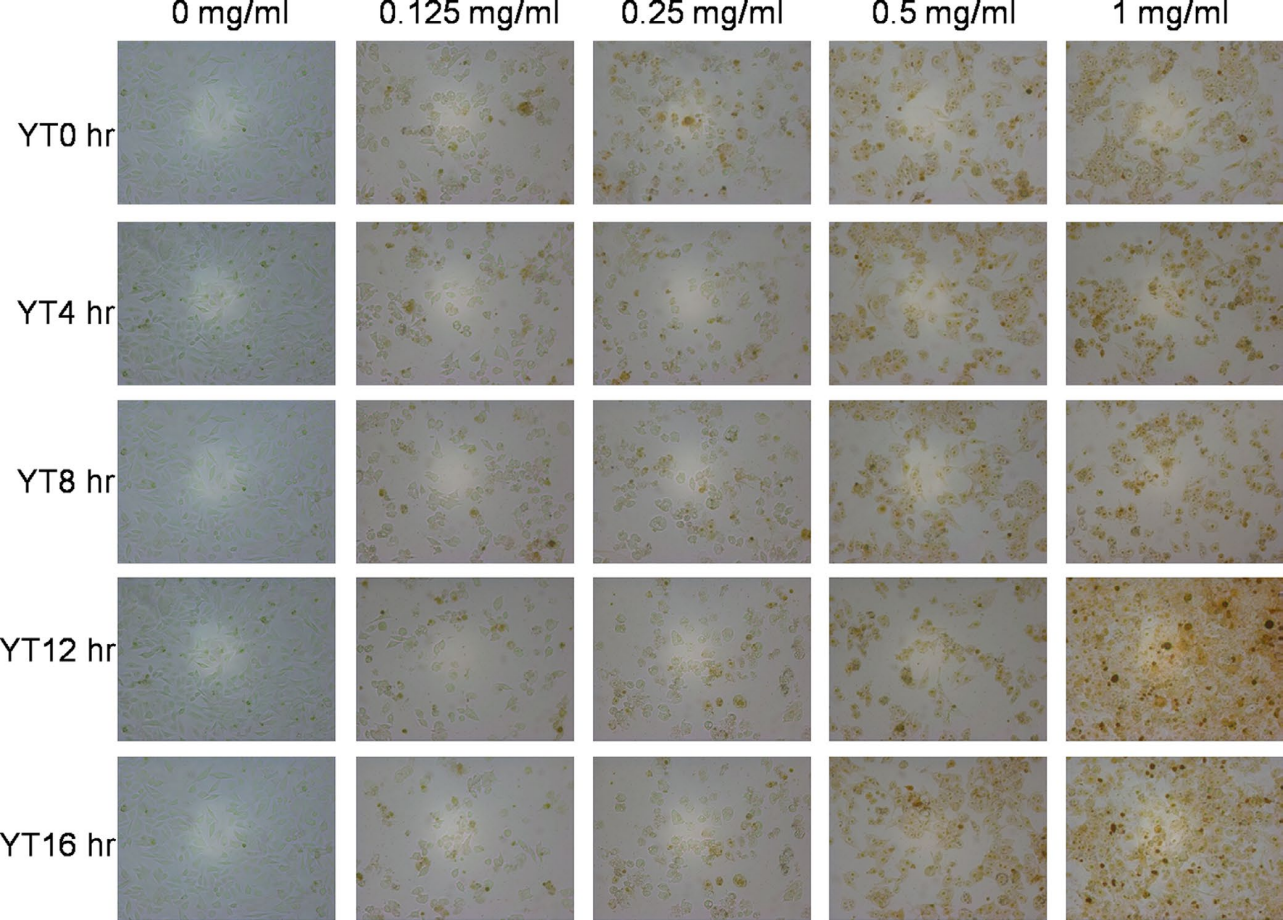
Representative pictures of HepG2 cells 24 hr after treatment with 0, 0.125, 0.25, 0.5, and 1 mg/ml EYTs

**Figure 3:**
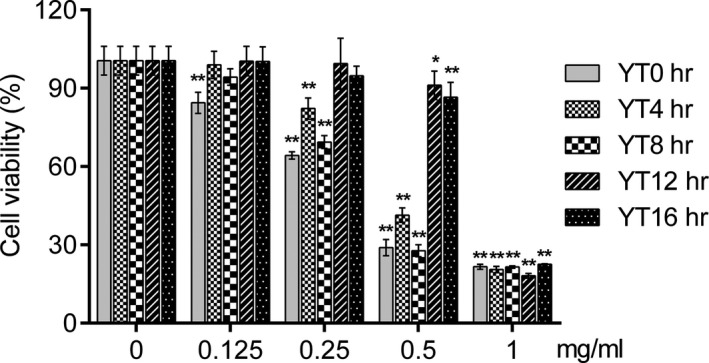
MTT assay results showing viability of HepG2 24 hr after treatment with 0, 0.125, 0.25, 0.5, and 1 mg/ml of each EYT. Data are presented as means ± *SD* of four independent experiments. *p < 0.05 versus the control group (0 mg/ml); **p < 0.01 versus the control group (0 mg/ml)

The cytotoxicity of EYTs on HepG2 was evaluated in terms of both viability and morphology. As shown in Figure 2, EYTs significantly changed the shape of HepG2 cells in a dose‐dependent manner, the nucleus of which gets shrinking and rounding and separates from its cytoplasm, with most potent effects seen with 1 mg/ml EYTs over 24 hr. In addition, HepG2 cells treated with the extracts showed altered membrane surface and loss of cellular adhesion. Furthermore, the MTT assay showed that EYTs significantly reduced HepG2 cell viability in a dose‐dependent manner after 24‐hr exposure (Figure 3) but had no influence to normal liver cells (Figure S1). The maximum inhibition was observed with 1 mg/ml EYTs. However, although the cytotoxic effects of YT12h samples were slightly higher compared to the other samples, the yellowing time had no significant effect on the antineoplastic activity of EYTs. Based on these results, we subsequently treated the HepG2 cells with 1 mg/ml EYTs for 24 hr.

### 3.5 EYTs inhibit HepG2 cell growth via the PI3K/AKT pathway and Bax/Bcl‐2

**Figure 4:**
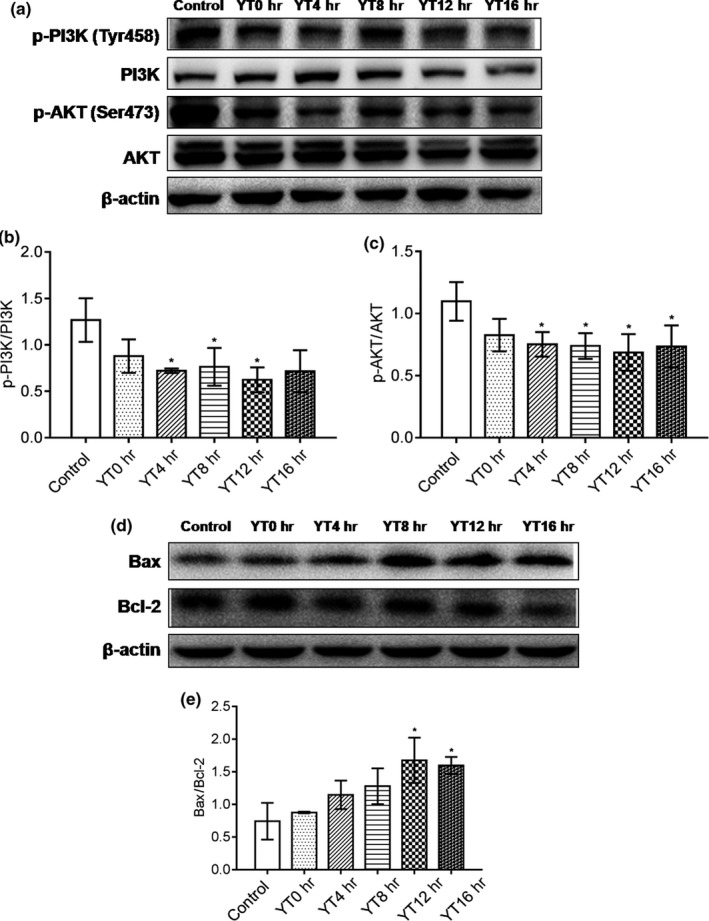
Representative immunoblots showing levels of p‐PI3K, PI3K, p‐AKT, and AKT in EYT‐treated HepG2 cells (a), expression of p‐PI3K relative to PI3K level (b), expression of p‐AKT relative to AKT level (c), immunoblots showing levels of Bax and Bcl‐2 in HepG2 cells (d), and expression of Bax relative to Bcl‐2 level (e). Data are presented as mean ± *SD* of three independent experiments. * *p* < 0.05 versus the control group; ** *p* < 0.01 versus the control group

To determine the molecular mechanism underlying the cytotoxic effect of EYTs, we analyzed the levels of proliferation and apoptosis‐related proteins in HepG2 cells treated with the EYTs. As shown in Figure 4a,b, p‐PI3K was significantly reduced by YT4h, YT8h, and YT12h (*p* < 0.05), with the most potent downregulation by YT12h. In addition, p‐AKT levels were also significantly reduced by YT4h, YT8h, YT12h, and YT16h extracts (*p* < 0.05; Figure 4a,c). Finally, YT12h and YT16h significantly elevated the Bax/Bcl‐2 ratio in HepG2 cells (Figure 4d,e; *p* < 0.05). Taken together, EYTs inhibit HepG2 cell growth by blocking proliferation and inducing apoptosis.

### 3.6 Correlation between yellowing time and biological activities

There was no significant correlation between the biological activities of EYTs and the yellowing time (*p* > 0.05; see Table 4).

**Table 4:** Correlation coefficient (*r*) between yellowing time and biological activities of Yinghong NO.9 yellow tea

	DPPH	OH	ABTS	MTT
Correlation coefficient (*r*)	0.452	0.801	0.311	0.859

## 4 DISCUSSION

Tea, made from the leaves of *Camellia sinensis*, is a popular beverage worldwide, owing to its significant health benefits (Xu et al., 2018). Based on the processing techniques and characteristics, tea can be broadly classified as green, white, yellow, black, oolong, and dark. The yellow tea is unique to China, dating back to the Tang Dynasty. However, as studies have highlighted the benefits of drinking tea in recent years, yellow tea has gained more popularity.

The processing steps for yellow tea are largely similar to those of green tea, with an additional steaming or “yellowing” step (Xu et al., 2018). The heat and microbial activity of this step result in chlorophyll destruction, polyphenol oxidation and isomerization, starch hydrolysis, and protein decomposition (Chen, Zhou, & Wang, 2012; Gong, Cai, Cai, & Jin, 2000; Xu et al., 2018), which bestow the special sensory characteristics of mellow flavor and bright yellow color. The biochemical characteristics of yellow tea are dependent on its processing parameters, especially the yellowing duration. Gong et al found that the decomposition of tea polyphenols and catechins was accelerated with increasing yellowing time, whereas the amino acid content increased during the initial stages and decreased after 9 hr of yellowing (Gong et al., 2000). Consistent with this, we found that the amino acid and sugar contents increased rapidly within the first 12 hr of yellowing, and more slowly thereafter. The spike in amino acid levels during yellowing is mainly attributed to the hydrolysis, pyrolysis, and oxidation of proteins (Wang & Li, 2006). The increased amino acids and sugars seen with longer processing of the Yinghong NO.9 yellow tea could be due to its high water content. However, the amino acid content eventually declined with prolonged yellowing, which might be responsible for the enhanced aroma. Interestingly, the content of polyphenols declined significantly during yellowing in a time‐dependent manner, consistent with the findings of Zhou et al. The total catechin levels decreased during the yellowing process, specifically with a progressive decline in EGCG, EGC, and ECG and increase in C and EC. The likely reason for these changes is the oxidation, isomerization, and thermal cracking of the more complex catechins like EGCG and ECG to simple catechins during the yellowing process (Friedman et al., 2009; Narumi et al., 2014; Zhou, Chen, Sun, Yuan, & Ni, 2005). Taken together, yellowing alters the composition of tea and may therefore affect its bioactivity as well.

In the normal process of life activities, the body will produce reactive oxygen species and free radicals, and their high content in the body will cause oxidative stress in the body, which is related to a variety of diseases such as cardiovascular disease and cancer. Antioxidants are widely concerned for their ability to react with unstable free radicals to protect the body from oxidative stress. At present, according to different detection mechanisms, a variety of detection methods are used to detect the antioxidant activity of antioxidants. We analyzed the scavenging ability to different free radicals of the yellow tea samples with different yellowing times. The results showed that although green tea (i.e., YT0h) showed better scavenging of DPPH, OH, and ABTS compared to yellow tea, the latter also exhibited potent antioxidative action in a concentration‐dependent manner, with maximum effects seen in samples yellowed for 12 and 16 hr. However, the yellowing time did not have a significant impact on the ability of EYTs to scavenge free radicals. In contrast, the level of FRAP, an indicator of total antioxidant effect, was significantly improved after yellowing for 16 hr. The strong antioxidant capacity of tea leaf extracts is well established. Gramza‐Michałowska et al showed a high antioxidative potential of yellow tea leaves on account of their high polyphenol content (Gramza‐Michałowska et al., 2016). Since the polyphenols decreased in the Yinghong NO.9 yellow tea with prolonged yellowing, its free radical scavenging ability was slightly inhibited. Taken together, the antioxidant potential of yellow tea is not diminished due to yellowing and may even be enhanced depending on the tea.

Tea extracts also have potential antineoplastic effects and are increasingly being investigated as alternative therapeutic agents against cancer. Therefore, we also analyzed the cytotoxic effects of the EYTs on a HepG2 liver cancer cell model and found that 0.5 mg/ml of the extract inhibited cell viability by 70%. In addition, the yellowing time did not have a significant impact on the cytotoxic effects of the EYTs. YT12h and YT16h showed slightly less inhibitory effects, likely due to the lower polyphenol content. Apoptosis induction is a key mechanism to kill cancer cells (Gupta, Varma, & Khandelwal, 2007; Yuan et al., 2012; Zhang, Wang, Zhu, Xu, & Ding, 2014), and several apoptosis‐related pathways have been harnessed to develop novel anticancer drugs. The PI3K/AKT signaling pathway is a prototypic survival pathway that plays a crucial role in promoting tumor cell growth and inhibiting apoptosis (Ao, Guan, Wang, & Wang, 2018; Gupta et al., 2007), and is constitutively activated in various cancer cells (Sun, Wang, Liu, & Wang, 2015; Sun, Zhang, et al., 2015). In addition, it is a key regulator of cancer cell growth, proliferation, and cell cycle (Franke, Hornik, Segev, Shostak, & Sugimoto, 2003; Liao, Zhang, Zhao, & Liu, 2018; Zhao et al., 2017), and therefore, a potential therapeutic target (Hamidi et al., 2017; Yuan et al., 2012).

The EYTs significantly inhibited the phosphorylation and activation of PI3K, as well as its downstream protein AKT. The Bax and Bcl‐2 proteins play a crucial role in the mitochondrial apoptotic pathway (Garcia‐Delgado, Valdés‐Sánchez, Calado, Diaz‐Corrales, & Bhattacharya, 2018; Zhao, Fu, Sun, & Liu, 2018), and the ratio of Bax/Bcl‐2 is a useful indicator of apoptosis (Del Principe et al., 2016; Khodapasand, Jafarzadeh, Farrokhi, Kamalidehghan, & Houshmand, 2015). In addition, apoptosis is also regulated by the opposing factions of the Bcl‐2 family (Marsden et al., 2002; Singh, Singh, Singh, Naqvi, & Singh, 2014; Zhao et al., 2017). Zhao et al found higher levels of the pro‐apoptotic Bax and lower levels of the anti‐apoptotic Bcl‐2 in HT‐29 cells treated with yellow tea extract compared to cells treated with the extracts of green tea (Zhao, 2009), indicating that the yellowing process may enhance the anticancer benefits. Consistent with this, the Bax expression in HepG2 cells treated with EYTs of varying yellowing times was slightly higher compared to the YT0h (green tea)‐treated cells, while that of Bcl‐2 was lower. Therefore, yellow tea might have a stronger anticancer effect than green tea.

## 5 CONCLUSIONS

To summarize, this is the first study to correlate the bioactivities of yellow tea with yellowing time. The extracts of yellow tea have potent antioxidant and antineoplastic effects. While the yellowing process enhanced the total antioxidant capacity of the EYTs, it did not affect their ability to kill hepatocarcinoma cells in vitro. Furthermore, the EYTs mediated their cytotoxic effects by inhibiting the prosurvival PI3K/AKT pathway and upregulating the Bax/Bcl‐2 ratio. In conclusion, yellow tea yellowing for 12 hr showed a strong bioactivity and anticancer ability, revealing the potential efficacy in inhibiting proliferation of cancer cells, but further studies are under way. Our findings not only supply scientific basis for the study of the relationship between yellow tea and health, but also provide new ideas for the development of high efficiency and safe therapeutic natural anticancer drugs.

## CONFLICT OF INTEREST

The authors declare that they do not have any conflict of interests.

## ETHICAL STATEMENTS

This study does not involve any human or animal testing.

## References

[fsn31036-bib-0001] Ao, R. , Guan, L. , Wang, Y. , & Wang, J. N. (2018). Silencing of COL1A2, COL6A3, and THBS2 inhibits gastric cancer cell proliferation, migration, and invasion while promoting apoptosis through the PI3k‐Akt signaling pathway. Journal of Cellular Biochemistry, 119(6), 4420–4434. 10.1002/jcb.26524 29143985

[fsn31036-bib-0002] Benzie, I. F. , & Strain, J. J. (1996). The ferric reducing ability of plasma (FRAP) as a measure of "antioxidant power": The FRAP assay. Analytical Biochemistry, 239(1), 70–76. 10.1006/abio.1996.0292 8660627

[fsn31036-bib-0003] Bian, L. , Yang, P. X. , Yao, Y. J. , Luo, Z. X. , Cai, X. M. , & Chen, Z. M. (2016). Effect of trap color, height, and orientation on the capture of yellow and stick tea thrips (Thysanoptera: Thripidae) and nontarget insects in tea gardens. Journal of Economic Entomology, 109(3), 1241–1248. 10.1093/jee/tow007 26842809

[fsn31036-bib-0004] Brand‐Williams, W. , Cuvelier, M. E. , & Berset, C. (1995). Use of a free radical method to evaluate antioxidant activity. Lebensm.‐Wiss. u.‐Technology, 28(1), 25–30. 10.1016/S0023-6438(95)80008-5

[fsn31036-bib-0005] Cabrera, C. , Giménez, R. , & López, M. C. (2003). Determination of tea components with antioxidant activity. Journal of Agricultural and Food Chemistry, 51(15), 4427–4435. 10.1021/jf0300801 12848521

[fsn31036-bib-0006] Chen, B. , Liao, J. , Hsu, A. , Tsai, P. , & Hsueh, C. (2018). Exploring optimal supplement strategy of medicinal herbs and tea extracts for bioelectricity generation in microbial fuel cells. Bioresource Technology, 256, 95–101. 10.1016/j.biortech.2018.01.152 29433051

[fsn31036-bib-0007] Chen, L. , Zhou, Y. , & Wang, Z. (2012). Research on the effect of yellowing with piling process on the quality of yellow tea. Tea Communication, 39, 8–11.

[fsn31036-bib-0008] Del Principe, M. I. , Bo, M. D. , Bittolo, T. , Buccisano, F. , Rossi, F. M. , Zucchetto, A. , … Del Poeta, G. (2016). Clinical significance of bax/bcl‐2 ratio in chronic lymphocytic leukemia. Haematologica, 101(1), 77–85. 10.3324/haematol.2015.131854 26565002 PMC4697894

[fsn31036-bib-0009] Franke, T. F. , Hornik, C. P. , Segev, L. , Shostak, G. A. , & Sugimoto, C. (2003). PI3K/Akt and apoptosis: Size matters. Oncogene, 22(56), 8983–8998. 10.1038/sj.onc.1207115 14663477

[fsn31036-bib-0010] Friedman, M. , Levin, C. E. , Lee, S. U. , & Kozukue, N. (2009). Stability of green tea catechins in commercial tea leaves during storage for 6 months. Journal of Food Science, 74(2), H47–H51. 10.1111/j.1750-3841.2008.01033.x 19323750

[fsn31036-bib-0011] Garcia‐Delgado, A. B. , Valdés‐Sánchez, L. , Calado, S. M. , Diaz‐Corrales, F. J. , & Bhattacharya, S. S. (2018). Rasagiline delays retinal degeneration in a mouse model of retinitis pigmentosa via modulation of Bax/Bcl‐2 expression. CNS Neuroscience & Therapeutics, 24(5), 448–455. 10.1111/cns.12805 29372592 PMC6489995

[fsn31036-bib-0012] Gong, Y. X. , Cai, L. W. , Cai, S. W. , & Jin, H. J. (2000). Study on the effect of stack‐cover process on the taste of yellow tea. Journal of Tea Science, 20, 110–113.

[fsn31036-bib-0013] Gramza‐Michałowska, A. , Kobus‐Cisowska, J. , Kmiecik, D. , Korczak, J. , Helak, B. , Dziedzic, K. , & Górecka, D. (2016). Antioxidative potential, nutritional value and sensory profiles of confectionery fortified with green and yellow tea leaves (*Camellia sinensis*). Food Chemistry, 211, 448–454. 10.1016/j.foodchem.2016.05.048 27283654

[fsn31036-bib-0014] Gupta, D. , Varma, S. , & Khandelwal, R. L. (2007). Long‐term effects of tumor necrosis factor‐α treatment on insulin signaling pathway in HepG2 cells and HepG2 cells overexpressing constitutively active Akt/PKB. Journal of Cellular Biochemistry, 100(3), 593–607. 10.1002/jcb.21080 16960890

[fsn31036-bib-0015] Hamidi, A. , Song, J. , Thakur, N. , Itoh, S. , Marcusson, A. , Bergh, A. , … Landström, M. (2017). TGF‐β promotes PI3K‐AKT signaling and prostate cancer cell migration through the TRAF6‐mediated ubiquitylation of p85α. Science Signaling, 10(486), eaal4186 10.1126/scisignal.aal4186 28676490

[fsn31036-bib-0016] Khodapasand, E. , Jafarzadeh, N. , Farrokhi, F. , Kamalidehghan, B. , & Houshmand, M. (2015). Is Bax/Bcl‐2 ratio considered as a prognostic marker with age and tumor location in colorectal cancer? Iran Biomedical Journal, 19, 69–75. 10.6091/ibj.1366.2015 25864810 PMC4412916

[fsn31036-bib-0017] Kmiecik, D. , Gramza‐Micha Owska, A. , & Korczak, J. (2018). Anti‐polymerization activity of tea and fruits extracts during rapeseed oil heating. Pharmaceutical Biology, 239, 858–864. 10.1016/j.foodchem.2017.07.025 28873644

[fsn31036-bib-0018] Kujawska, M. , Ewertowska, M. , Adamska, T. , Ignatowicz, E. , Gramza‐Michałowska, A. , & Jodynis‐Liebert, J. (2016). Protective effect of yellow tea extract on N‐nitrosodiethylamine‐induced liver carcinogenesis. Pharmaceutical Biology, 54, 1891–1900. 10.3109/13880209.2015.1137600 26839940

[fsn31036-bib-0019] Liao, Y. X. , Zhang, Z. P. , Zhao, J. , & Liu, J. P. (2018). Effects of fibronectin 1 on cell proliferation, senescence and apoptosis of human glioma cells through the PI3K/AKT signaling pathway. Cellular Physiology and Biochemistry, 48, 1382–1396. 10.1159/00049209 30048971

[fsn31036-bib-0020] Marsden, V. S. , O'Connor, L. , O'Reilly, L. A. , Silke, J. , Metcalf, D. , Ekert, P. G. , … Strasser, A. (2002). Apoptosis initiated by Bcl‐2‐regulated caspase activation independently of the cytochrome c/Apaf‐1 caspase‐9 apoptosome. Nature, 419(6907), 634–637. 10.1038/nature01101 12374983

[fsn31036-bib-0021] Narumi, K. , Sonoda, J.‐I. , Shiotani, K. , Shigeru, M. , Shibata, M. , Kawachi, A. , … Motoya, T. (2014). Simultaneous detection of green tea catechins and gallic acid in human serum after ingestion of green tea tablets using ion‐pair high‐performance liquid chromatography with electrochemical detection. Journal of Chromatography B, 945–946, 147–153. 10.1016/j.jchromb.2013.11.007 24342507

[fsn31036-bib-0022] Ng, D. , Coventry, K. , Lim, P. , Wiltshire, J. , Qiao, G. G. , Boulton, A. , & Senior, G. (2009). An improved technique for concentration measurement of galactomannan solutions by differential refractive index. Carbohydrate Polymers, 77(1), 150–153. 10.1016/j.carbpol.2008.12.028

[fsn31036-bib-0023] Singh, B. N. , Rawat, A. K. , Bhagat, R. M. , & Singh, B. R. (2017). Black tea: Phytochemicals, cancer chemoprevention, and clinical studies. Critical Reviews in Food Science and Nutrition, 57(7), 1394–1410. 10.1080/10408398.2014.994700 26561007

[fsn31036-bib-0024] Singh, B. N. , Singh, H. B. , Singh, A. , Naqvi, A. H. , & Singh, B. R. (2014). Dietary phytochemicals alter epigenetic events and signaling pathways for inhibition of metastasis cascade. Cancer and Metastasis Reviews, 33(1), 41–85. 10.1007/s10555-013-9457-1 24390421

[fsn31036-bib-0025] Sun, Z. G. , Wang, Z. , Liu, X. Y. , & Wang, D. (2015). New development of inhibitors targeting the PI3K/AKT/mTOR pathway in personalized treatment of non‐small‐cell lung cancer. Anti‐Cancer Drugs, 26(1), 1838–14. 10.1097/CAD.0000000000000172 25304988

[fsn31036-bib-0026] Sun, L. I. , Zhang, S. , Yu, C. , Pan, Z. , Liu, Y. , Zhao, J. , … Li, Y. (2015). Hydrogen sulfide reduces serum triglyceride by activating liver autophagy via the AMPK‐mTOR pathway. American Journal of Physiology‐Endocrinology and Metabolism, 309(11), E925–E935. 10.1152/ajpendo.00294.2015 26442880

[fsn31036-bib-0027] Tang, Y. Z. , & Liu, Z. Q. (2007). Free‐radical‐scavenging effect of carbazole derivatives on DPPH and ABTS radicals. Journal of the American Oil Chemists Society, 84(12), 1095–1100. 10.1007/s11746-007-1149-y

[fsn31036-bib-0028] Teng, Y. , Li, D. , Guruvaiah, P. , Xu, N. , & Xie, Z. (2018). Dietary supplement of large yellow tea ameliorates metabolic syndrome and attenuates hepatic steatosis in db/db mice. Nutrients, 10(1), 75 10.3390/nu10010075 PMC5793303 29329215

[fsn31036-bib-0029] Troll, W. , & Cannan, R. K. (1953). A modified photometric ninhydrin method for the analysis of amino and imino acids. Journal of Biological Chemistry, 200, 803–811. 13034841

[fsn31036-bib-0030] Wang, Y. , & Li, C. (2006). Studies on the variation of amino acid content and affecting factors in quality tea. Southwest China Journal of Agricultural Sciences, 6, 1121–1126.

[fsn31036-bib-0031] Xu, J. , Wang, M. , Zhao, J. , Wang, Y.‐H. , Tang, Q. , & Khan, I. A. (2018). Yellow tea (*Camellia sinensis* L.), a promising Chinese tea‐processing, chemical constituents and health benefits. Food Research International, 107, 567–577. 10.1016/j.foodres.2018.01.063 29580521

[fsn31036-bib-0032] Yuan, L. , Wang, J. , Xiao, H. F. , Xiao, C. X. , Wang, Y. T. , & Liu, X. B. (2012). Isoorientin induces apoptosis through mitochondrial dysfunction and inhibition of PI3K/Akt signaling pathway in HepG2 cancer cells. Toxicology and Applied Pharmacology, 265(1), 83–92. 10.1016/j.taap.2012.09.022 23026832

[fsn31036-bib-0033] Zhang, L. , Wang, H. D. , Zhu, J. D. , Xu, J. G. , & Ding, K. (2014). Mollugin induces tumor cell apoptosis and autophagy via the PI3K/AKT/mTOR/p70S6K and ERK signaling pathways. Biochemical and Biophysical Research Communications, 450(1), 247–254. 10.1016/j.bbrc.2014.05.101 24887566

[fsn31036-bib-0034] Zhao, J. , Zhu, J. B. , Lv, X. S. , Xing, J. S. , Liu, S. , Chen, C. , & Xu, Y. H. (2017). Curcumin potentiates the potent antitumor activity of ACNU against glioblastoma by suppressing the PI3K/AKT and NF‐kB/COX‐2 signaling pathways. Onco Targets and Therapy, 10, 5471–5482. 10.2147/OTT.S149708 PMC5695266 29180881

[fsn31036-bib-0035] Zhao, T. , Fu, Y. , Sun, H. , & Liu, X. (2018). Ligustrazine suppresses neuron apoptosis via the Bax/Bcl‐2 and caspase‐3 pathway in PC12 cells and in rats with vascular dementia. International Union of Biochemistry and Molecular Biology, 70(1), 60–70. 10.1002/iub.1704 29247598

[fsn31036-bib-0036] Zhao, X. (2009). In vitro anticancer effect of yellow tea in HT‐29 human colon cancer cells. Journal of Beijing Union University (Natural Sciences), 3, 11–13.

[fsn31036-bib-0037] Zhou, J. , Chen, Y. , Sun, Y. , Yuan, F. , & Ni, D. (2005). Change of quality of Luyuan yellow tea during processing. Journal of Huazhong Agricultural University, 1, 88–92.

[fsn31036-bib-0038] Zhou, Y. , Zeng, L. , Liu, X. , Gui, J. , Mei, X. , Fu, X. , … Yang, Z. (2017). Formation of (E)‐nerolidol in tea (*Camellia sinensis*) leaves exposed to multiple stresses during tea manufacturing. Food Chemistry, 231, 78–86. 10.1016/j.foodchem.2017.03.122 28450026

