# Supplementary material for: Effect of yellowing time on bioactive compounds in yellow tea and their antiproliferative capacity in HepG2 cells
Source: Food Sci Nutr. 2019 Apr 18;7(5):1838–47. doi: 10.1002/fsn3.1036 (PMC6526664; doi:10.1002/fsn3.1036)
Supplement: Supplementary file 1 [file FSN3-7-1838-s001.docx]

**Supplementary Materials:**

**Figure S1.** MTT assay results showing viability of LO2 normal liver cells 24 h after treatment with 0, 0.125, 0.25, 0.5 and 1 mg/ml of each EYTs.

**
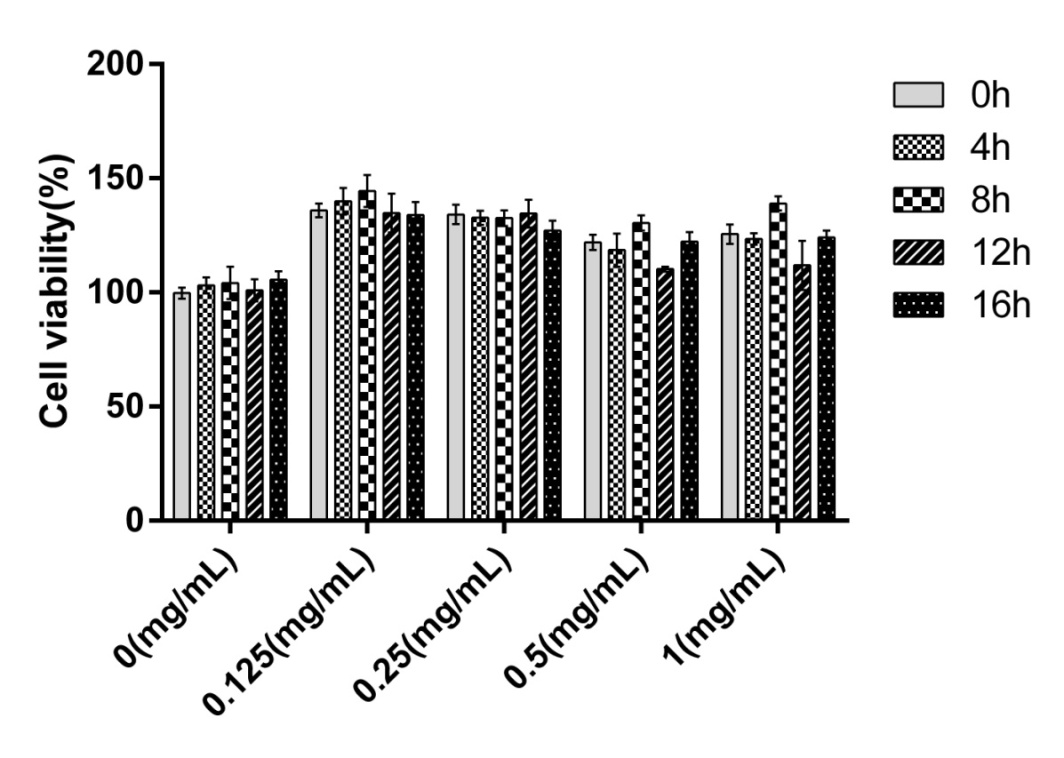
**
